# Dual wavelength retinal vessel oximetry – influence of fundus pigmentation

**DOI:** 10.1038/s41433-022-02325-7

**Published:** 2022-12-02

**Authors:** Katrin Hirsch, Robert P. Cubbidge, Rebekka Heitmar

**Affiliations:** 1 The University of the West of England, School of Health and Social Wellbeing, Glenside Campus, Bristol, BS16 1DD UK; 2 ABDO College, Godmersham Park, Canterbury, Kent CT4 7DT UK; 3 Aston University, Aston Triangle, Birmingham, B4 7ET UK; 4 University of Huddersfield, School of Applied Sciences, Centre for Vision across the Life Span, Queensgate, HD1 3DH UK

**Keywords:** Prognostic markers, Prognostic markers

## Abstract

**Background:**

Clinical methods examining oxygenation parameters in humans have been used in many different care settings, but concerns have been raised regarding their clinical utility when assessing people with darker skin pigmentation. While saturation values can be crucial in emergency medicine, they are equally valuable in assessing disease mechanisms and monitoring change in disease progression. Retinal pigmentation varies across individuals and hence, can impact on retinal oxygen parameters. The objective of this study was to quantify and eliminate the impact of retinal pigmentation on retinal vessel oxygen saturation parameters measured in the superficial retinal arterioles and venules.

**Methods:**

105 healthy individuals of varying skin colour, iris colour and heritage were included. Following a full eye exam to exclude any ocular abnormality, all participants underwent intraocular pressure, systemic blood pressure measurements and dilated dual wavelength retinal photography. Rotation matrices were employed to minimise the dependency of retinal pigmentation on arterial and venous oxygen saturation measurements determined in a concentric measurement annulus.

**Results:**

Retinal oxygen saturation in venules showed a linear correlation with retinal pigmentation (*y* = 0.34 × *x* + 38.598), whereas arterial saturation followed a polynomial pattern (*y* = 0.0089 × *x*^2^ + 0.7499 × *x* *+* 85.073). Both arterial and venous saturation values were corrected using local fundus pigmentation. Pre-correction retinal arterial and venous oxygen saturation were 89.0% (±13.1) and 43.7% (±11.5), respectively, and post- correction values were 94.8% (±8.7) for arteries and 56.3% (±7.0) veins.

**Conclusions:**

When assessing multi-ethnic cohorts, it is important to consider the impact of pigmentation on imaging parameters and to account for it prior to clinical interpretation.

## Introduction

Dual-wavelength retinal vessel oximetry has become a promising ocular imaging modality to assess and study the mechanisms involved in ocular vascular conditions such as glaucoma [1–3], diabetes mellitus (DM) [4–7], age-related macular degeneration (AMD) [8, 9] and retinal vessel occlusions [10–13]. The ability to non-invasively measure the oxygen saturation in the superficial retinal vasculature (referred to as arterial SO2) as well as the amount that is drained via the superficial retinal venous circulation (referred to as venous SO2) can provide a surrogate measure of oxygen metabolism [14, 15]. Retinal oximetry, therefore, maybe capable to provide further insight into hypoxia and hyperoxia of retinal tissue.

The measurement principle is based on a modification of the Beer–Lambert Law (see Eq. (1)), whereby the optical densities of the retinal vessels and its surrounding tissue are measured [16].

1 $$A = log_{10}I_{out} = \varepsilon \, \times I \, \times c$$

Equation (1): The Beer–Lambert Law

As it is not possible to directly measure the light being absorbed and transmitted through the blood vessel, a modified version of the Beer–Lambert law allows an approximation to determine the oxygen content in retinal vessels by measuring the optical density of the retinal blood vessels (I_in_) and relate this to the optical density obtained from neighbouring tissue (I_out_) [17]. Equation (2) details how to calculate the oxygen saturation (SO2) based on optical density measurements (ODR, see Eq. (3)). Here, the optical densities of two distinct wavelengths, one oxygen sensitive (610 nm) and one oxygen insensitive (548 nm), are measured.

2 $$SO_2 = 100\% - \left( {\frac{{ODR - ODR_{a,100}}}{{OS}}} \right) - (a - VD) \times b + \left( {c - \log {\textstyle{{I\frac{{610}}{{out}}} \over {I\frac{{548}}{{out}}}}}} \right) \times d$$

Equation (2): Algorithm to calculate SO2 values based on optical density ratio (ODR) [17]. whereby

3 $$ODR = \frac{{\log \frac{{I\frac{{610}}{{out}}}}{{I\frac{{610}}{{in}}}}}}{{\log \frac{{I\frac{{610}}{{out}}}}{{I\frac{{548}}{{in}}}}}}$$

Equation (3): Algorithm to determine the optical density ratio (ODR) from measured intensities (I); ‘OUT’ refers to measurements at neighbouring tissue level; ‘IN’ refers to measurements taken at vessel plane; 610 and 548 refers to wavelengths used for measurement

Additionally, *OS* is 0.0023/%SO2, *ODR*_*a,100*_ is 0.01357 and *VD* is the vessel diameter.

The experimentally derived constants *a, b, c* and *d* are different for arteries and veins, and refer to 109 μm, 0.0667% SO2/μm, 0.265 and 15.149% SO2 for arteries and 125 μm, 0.2626% SO2/μm, 0.272 and 51.055% SO2 for veins [17].

This method is not without limitations since it does not measure light leaving the blood vessel directly. The use of reflectivity rather than absorption is influenced by media opacites [18–20], reflections of light along and across a given vessel section not being homogenous and depending on vessel size (VD), retinal curvature and blood velocity [17, 21, 22].

More importantly, saturation values may also vary depending on retinal pigmentation [17, 21]. Beach et al. found that reflectivity of retinal tissue depends upon the wavelength used for measurement [21]. Hammer et al. also found a significant influence of retinal pigmentation for venous but not for arterial SO2. They corrected for retinal pigmentation accounting for surrounding tissue reflectance properties (see Supplementary Table S1 for details [17, 23–27]). Both studies used different methods to classify pigmentation, which makes direct comparisons difficult. The studies used hair and iris colour as surrogate measures for retinal pigmentation. Whilst both classifications were of categorical nature, Beach grouped according to hair and iris colour, whereas Hammer et al. stratified a group of Caucasians (CA) only using iris colour (blue, grey, green, brown). In addition, both publications have limited sample sizes: The study by Beach et al. only assessed seven individuals, while Hammer et al. analysed 20 individuals. A more recent study comparing the retinal vessel oxygen saturation measurements of 20 young healthy CA and 20 South Asian (SA) individuals found a statistically significant difference in saturation values between the two ethnic groups [28]. These findings suggest that pigmentation compensation parameters within the software algorithm do not sufficiently compensate for the effect of retinal pigmentation on the determination of SO2. The aim was to identify a method to account and correct oxygen saturation parameters obtained by dual wavelength retinal oximetry to obtain pigmentation independent saturation values which can be used in multi-ethnic cohorts with a wide range of iris colours and skin pigmentation.

## Methods

Following favourable opinion from the Aston University Ethics Committee (Ref 986; compliant with the Declaration of Helsinki) we recruited 105 otherwise healthy individuals (41 men and 64 women) of different ethnic backgrounds and varying iris colour (see Table 1) for sample characteristics. All subjects were recruited by inviting Aston University students, staff and other volunteers.

**Table 1:** IOP is intraocular pressure; SBP is systolic blood pressure; DBP is diastolic blood pressure.

*N* = 105	Mean (SD)
Age [years]	27 (10)
IOP [mmHg]	14 (3)
SBP [mmHg]	112 (11)
DBP [mmHg]	72 (8)
Axial length [mm]	24.4 (1.5)
Iris colour score (reference 25)	15.2 (7.7); range 1–24

Ethnicity		Iris colour score
	*n*	Median, range
Caucasian	50	9, 1–23
South Asian	38	22, 17–24
Mediterranean	6	17, 5–17
East Asian	5	21, 18–24
Black	4	22, 15–23
Arab	2	20; 3

**Fig. 1:**
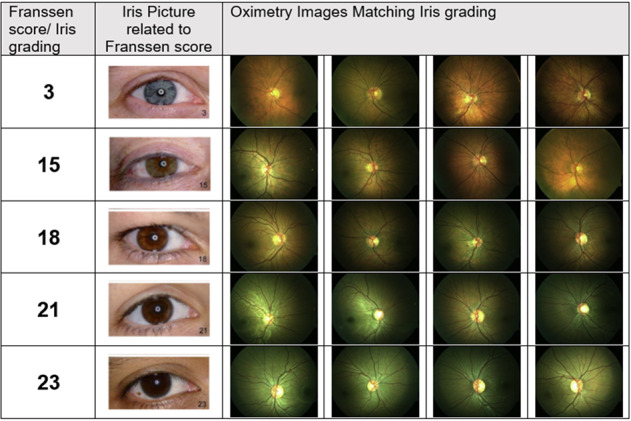
Participant samples for a range of iris gradings. Examples of participants retinal images obtained with the retinal oximeter and their accompanying iris grading according to Franssen [29].

Exclusion criteria for the study were intraocular pressure (IOP) > 24 mmHg, hypertension (systolic blood pressure (SBP) >140 mmHg and diastolic blood pressure (DBP) > 90 mmHg), any systemic or ocular vascular condition, previous refractive surgery, and previous ocular trauma. All participants were advised to abstain from caffeinated beverages for a minimum of 4 h prior to their scheduled appointment. After written informed consent was obtained, we measured axial length (IOLMaster, Zeiss Meditech, Germany) and IOP of all participants using a rebound tonometer (I-Care, Tiolat OY, Finland). Systemic blood pressure (BP) was assessed by calculating the mean of three measurements using a digital sphygmomanometer (UA767, A&D, PMS Instruments, Maidenhead) in a sitting position after resting for 15–20 min in a temperature-controlled room at 22 degree C. Iris colour was classified according to the iris colour scheme published by Franssen et al. [29]. Figure 1 details examples of fundus images from a selection of study participants along with their respective iris grading according to Franssen et al.

### Retinal oximetry

One unselected eye of each participant was dilated with one drop of tropicamide (tropicamide 1%, Minim, Bausch & Lomb). Once pupils were fully dilated, retinal images were captured using the IMEDOS Oxygen module consisting of a Zeiss F450^+^ fundus camera with a dual bandpass filter inserted in the illumination pathway (IMEDOS Systems, Jena, Germany) [17]. The system simultaneously captures images at one isosbestic wavelength (548 nm) and one oxygen sensitive wavelength (610 nm). All images were captured with the camera angle set at 50 degrees and the optic nerve head (ONH) centred [30]. To ensure stable measurements, three images of each participant were captured.

### Image analyses

**Fig. 2:**
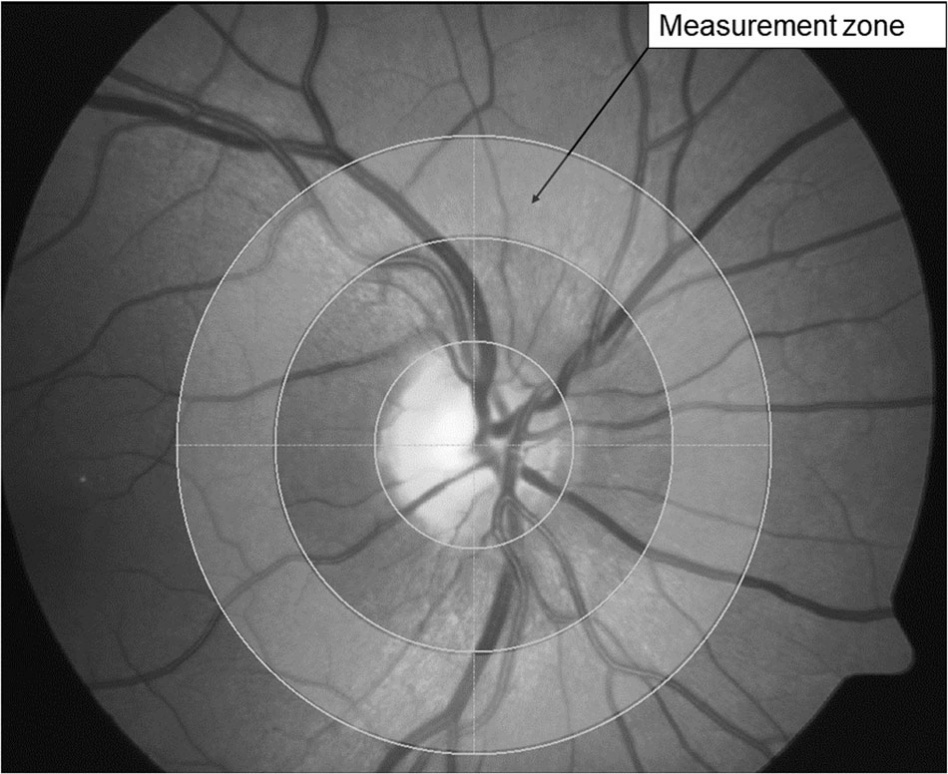
Measurement annulus. The shaded area in fundus image represents the concentric measurement annulus around the optic disc which is ½ disc diameter away from the optic nerve head margin and ½ disc diameter in width.

For image analysis the multi-measurement facility of the VesselMap software was used (IMEDOS, VesselMap version 2.82, Jena, Germany). The software allows for identical vessel segments being measured across multiple images for each participant, three images were analysed by including all vessels cursing through the measurement annulus around the ONH (the measurement annulus is a concentric ring, ½ disc diameter in width and ½ disc diameter distant from the ONH margin, see Fig. 1 for measurement annulus). Only vessels larger than 70 μm in diameter were included in the analyses [21, 31]. In addition, each included vessel segment had to have a minimum of ten valid saturation measurements, and the vessel diameter variance along the chosen vessel had to be less than 10%. In addition to optical density ratios, vessel diameters and saturation values, the VesselMap software also provides quantitative values for retinal pigmentation (RP) at each measured vessel section. Global retinal arteriolar and venular SO2 and pigmentation values were calculated (using the measured optical densities) by averaging the results from all assessed vessel segments measurement across the three images in the measurement annulus (see Fig. 2).

### Data analysis

Following normalisation of global retinal pigment (RP_i_%) using the equation

$$RP_i\% = 100 \, \times \, \frac{{RP_i}}{{RP_{{\it{max}}} - RP_{{\it{min}}}}}$$

the values for each data (‘i’) was plotted against arterial vessel saturation and against venous oxygen saturations to identify the nature of the relationships. To calculate RP independent saturation values, a rotation matrix (see Eq. (4)) was applied using the results from the best fit curves of the correlation results. For the relationship between arterial RP_i_% and arterial SO2 the best fit curve was *y* = 0.0089 × *x*^2^ + 0.7499 × *x* + 85.073; *y*′ = 2 × 0.0089 × *x* + 0.7499

4 $$\left[\begin{array}{c}\cos (Rt)\sin (Rt)\\ -\sin (Rt)\cos (Rt)\end{array}\right]{({{{\mathrm{a}}}})}\,{\mathrm {and}}\,\left[\begin{array}{c}\cos (Rt)-\sin (Rt)\\ \sin (Rt)\cos (Rt)\end{array}\right]{({{{\mathrm{b}}}})}$$

Equation (4): (a) refers to the clockwise data rotation matrix and (b) refers to anticlockwise data rotation matrix used to achieve pigmentation independent SO2 values.

For veins, the relationship of RP_i_% and SO2 values was of linear nature (*y* = 0.34 × *x* + 38.598). The data was rotated clockwise around the venous SO2 value y = 54% and x = 43.1 for RPi%, which corresponds to the apex of the BFC for arterial SO2 data. The rotation angle (Rt) for venous data was calculated as tan(Rt) which equates to the slope of the trendline and was 0.34.

### Statistical analyses

All SO2 data were tested for normal distribution prior to further analyses by using the Kolmogorov–Smirnov test. IBM SPSS version 28.0.1.0 (142) was used for normal distribution testing. Stepwise forward regression analysis was conducted to explore if ocular oxygen parameters were associated with ocular biometry and refraction. Data transformation to achieve pigmentation independent SO2 values for arteries and veins was carried out using Microsoft Excel. R^2^ analysis (Microsoft Excel) was used as measure of pigmentation dependency of SO2 values. A R^2^ of less than 0.14 was considered a ‘small’ dependency and a R^2^ greater or equal than 0.59 was considered ‘large’ dependency. Sample size was estimated for the assumption of a linear dependency and one independent variable according to the equation *N* = 104 + p, with p being the number of independent variables [32].

## Results

**Fig. 3:**
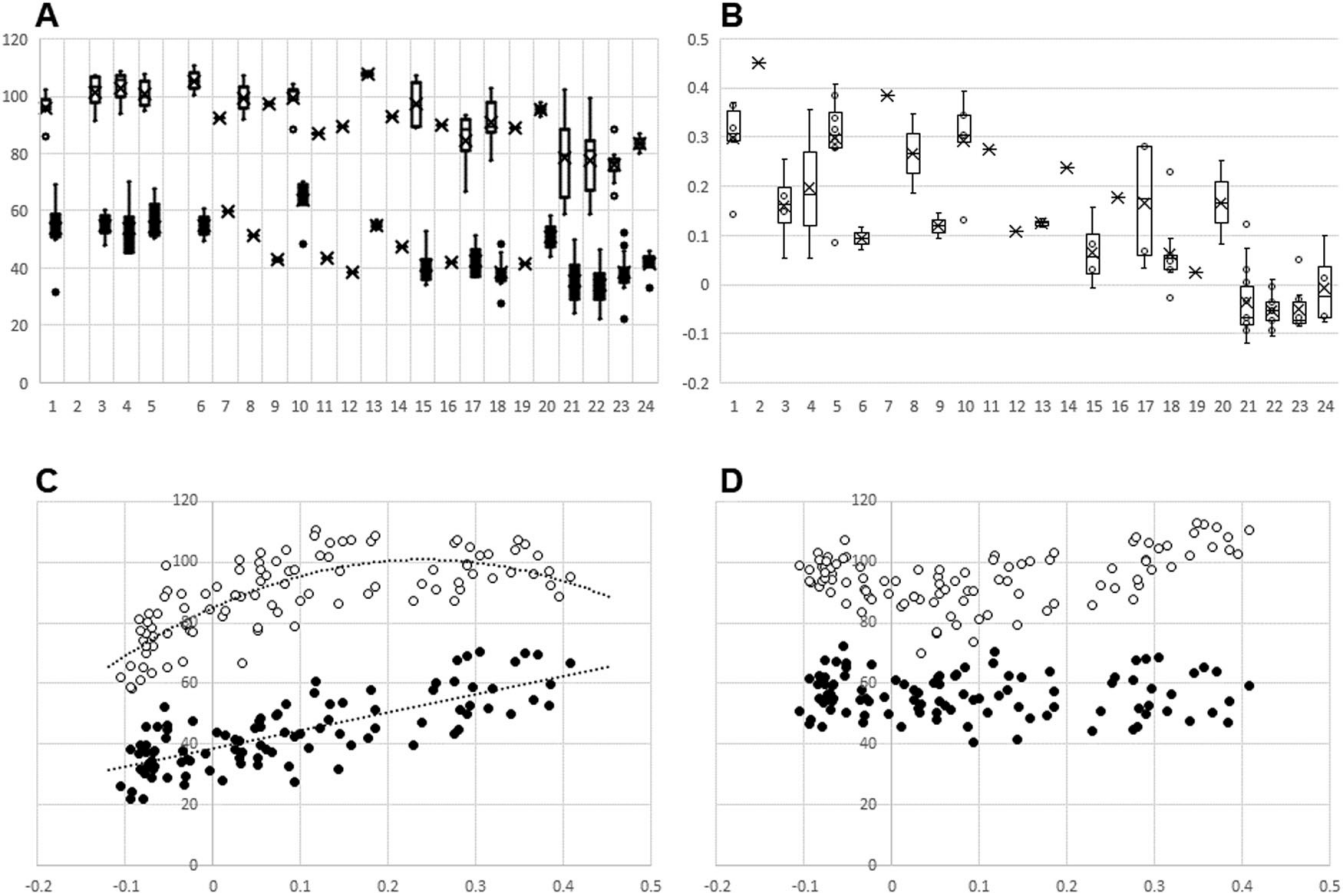
Graphical representation of data analyses. Unfilled circles denote arterial SO2; filled circles denote venous SO2; **A** SO2 data plotted against Franssen score; **B** RP plotted against Franssen score; **C** SO2 plotted against RP pre-correction; **D** SO2 plotted against RP post correction.

Demographic data of the sample can be found in Table 1. Arterial and venous SO2 data were normally distributed (D(104) = 0.077, p = 0.188 and D(104) = 0.075, p = 0.200 for arteries and veins, respectively). Retinal vessel oxygen saturation parameters were independent of ocular biometry and refractive parameters. Retinal oxygen saturation values decreased with increasing pigment score (i.e. Franssen score, see Fig. 3A), and increasing Franssen score increased with increasing retinal pigment as measured by dual wavelength photography (see Fig. 3B).

Retinal saturation values plotted against retinal pigmentation values of the surrounding tissue followed a polynomial pattern in retinal arteries (red data points), whereas the venular saturation (blue data points) decreased linearly with increasing levels of pigmentation (see Fig. 3C). Before data rotation, R^2^ for the correlation between arterial and venous SO2 with RP were 0.613 and 0.588, respectively. After data rotation R^2^ were 0.145 and 0.00 for arterial and venous SO2 vs RP relationships, respectively.

The mean SO2 results of the sample before data rotation were 89.0% (±13.1) and 43.7% (±11.5) for arteries and veins, respectively. Following correction for retinal pigmentation, retinal arterial and venous SO2 were 94.8% (±8.7) and 56.3% (±7.0).

## Discussion

To date, only a handful of studies explored retinal oxygen saturation parameters, and whether there is an impact due to different retinal pigmentation [17, 21, 33]. No study to date has been able to quantify retinal pigmentation, but rather used ethnicity and/or iris colour as a proxy to refer and discuss changes in retinal pigmentation [17, 21, 33]. The present study assessed a multi-ethnic sample of 105 healthy normotensive volunteers, and quantified their retinal pigmentation objectively using optical density measurements from the tissue surrounding the vessels in the measurement annulus (see Figs. 1 and 2) as well as classifying iris colour using a subjective scale [29]. Ethnic background alone is a poor marker of retinal pigmentation. Skin tone or iris colour alone exhibit limited ability to predict retinal pigmentation (see Fig. 3A) for iris colour relationship with SO2 measurements). In fact, several of the participants of South Asian descent exhibited light irises and several Caucasian participants exhibited dark irises and darker pigmented fundi than their South Asian counterparts with similar irises. However, iris colour is a better predictor than skin tone as it doesn’t vary due to seasonal changes [34]. Nevertheless, iris colour is difficult to classify as grading by visual inspection is subjective.

Uncorrected oxygen saturation values are affected by retinal pigmentation as dual wavelength retinal photography relies on reflectance values as described earlier. This paper shows that both, retinal arteriolar and venular SO2 values vary with iris and fundus pigmentation (see Fig. 1). However, the relationship between measured fundus pigmentation (RPi%) and arteriolar and venular SO2 exhibited different trends as can be seen from Fig. 3C. Venous SO2 showed a linear correlation with measured retinal pigmentation, arterial values showed an exponential relationship. This observed distribution of linear (venous SO2) and exponential (arterial SO2) nature in our sample may not be the same in other samples but is likely to vary, depending on pigmentation and ethnicity of a given sample. Neglecting this discrepancy may lead to artifactually low saturation values being recorded in those with darker fundus pigmentation irrespective of heritage, which in turn affects A-V calculations, which have been used as surrogate markers for oxygen consumption of the retinal tissue [15]. Studies that examined patients with ocular pathologies indicate increased SO2 levels in venules of patients suffering from glaucoma [1–3] as well as in diabetic retinopathy depending on its severity [6, 35]. Additionally, the increased SO2 has been shown to be related with decreased visual function in glaucoma [36, 37]. An erroneously low arterial SO2 value in individuals with darker fundi could mislead the observer, assuming tissue hypoxia. An erroneously low venous SO2 value in individuals with darker fundi on the other hand, may underestimate signs of tissue hypoxia.

Opposing to the study by Hammer and colleagues [17], which found a linear dependency between iris pigmentation and venous SO2 only, this research found a dependency of both, arteriolar and venular oxygen saturation and fundus pigmentation. The reason for this discrepancy is most likely due to the stratification scheme of the aforementioned study. Hammer et al. classified iris colour into three categories [17]. Moreover, iris colour may be a poor predictor of retinal pigmentation as darker pigmented individuals can have light coloured irises and vice versa (see Fig. 3A).

A study by Jani et al. used the same iris classification system as this study, but its sample size was smaller (*n* = 61) and did not cover the full range of the Franssen scores of iris pigmentation [33]. In addition, some of their participants were smokers and had controlled hypertension, which are both factors with a potential impact on oxygen saturation measurements.

The effect of pigmentation of the observed structure is not constant across the spectrum of wavelengths used for capturing the dual wavelengths oximetry images but effects the green and red channels differently. This effect can be seen in Fig. 1: Images of individuals with darker fundi show a greenish hue.

Peripheral oxygen saturation has been used in several clinical settings. During the Covid 19 pandemic, pulse oximetry has become a tool for monitoring patients with COVID 19 at home [38], not least due to its low cost and ease of use. Due to its more frequent use during the current Covid-19 pandemic, it rapidly became evident that skin pigmentation can impact its clinical decision making as it reported erroneous oxygen saturations in individuals with darker skin tones [39]. Cabanas and colleagues have collated a systematic review which highlights the influence of skin pigmentation on pulse oximetry, its accuracy, and implications in clinical decision [39]. The review highlights the larger variability in oximetry result occurring particularly at lower saturation levels and in cases with darker skin pigmentation. Furthermore, Cabanas and colleagues detail the lack of studies examining the impact of skin pigmentation on saturation values obtained by finger pulse oximeters. The same has been described by Garg and colleagues reviewing current retinal oximetry technologies [40]. This review paper emphasises that addressing the pigmentation dependency of retinal vessel oxygen parameter is paramount for the technique to become a suitable clinical tool especially when assessing multi-ethnic cohorts.

This research was limited by the restricted retinal location assessed (see Fig. 2), as we only evaluated the largest superficial retinal arteries and veins in a concentric annulus around the optic nerve head. For one individual, the effect of fundus pigmentation is not even across the retina. To account for this, the present study used a global measure of retinal pigmentation including all surrounding tissue pigmentary values of the vessels under observation in the predetermined measurement annulus.

In summary, this study proposes a method to correct retinal vessel oxygen saturation (as measured by dual wavelengths photography) in studies with multi-ethnic populations.

### Summary

#### What was known before

- Dual wavelength retinal oximetry results may depend upon retinal pigmentation.

#### What this study adds

- The study highlights the importance of considering retinal pigmentation in oximetry as results are influenced by retinal pigmentation values.

## Supplementary information

Supplemental Table

## Data Availability

The datasets generated during and/or analysed during the current study are available from the corresponding author on reasonable request.
