# Supplementary material for: Dual wavelength retinal vessel oximetry – influence of fundus pigmentation
Source: Eye (Lond). 2022 Dec 2;37(11):2246–51. doi: 10.1038/s41433-022-02325-7 (PMC9716545; doi:10.1038/s41433-022-02325-7)
Supplement: Supplementary file 1 — Supplemental Table [file 41433_2022_2325_MOESM1_ESM.docx]

| Sample/  Publication | Our sample [n=105] | Hammer et. al. 2008 (34)  [n=20] | Beach et. al. 1999 (23)  [n=5] | Geirsdottir et.al. 2012 (24)  [n=120] | Mohan et.al. 2015 (25)  [n=98] | Waizel et al. 2018 (26)  [n=118 eyes of 63 adults] | Nakano et. al. 2016 (27)  [n=252] | Yip et. al. 2014  (28)  [n=118] |
| --- | --- | --- | --- | --- | --- | --- | --- | --- |
| Oximeter | IMEDOS | IMEDOS | Experimental set up | OXYMAP | OXYMAP | IMEDOS | OXYMAP | OXYMAP |
| Ethnicity |  | Caucasian | Caucasian | Caucasian | Indian | Caucasian | Japanese | Not specified South Asian community |
| Arterial SO_2_  pre-correction [%] | 89.0 +/-13.1 |  |  | 92.2 +/-3.7 | 90.3 +/-6.6 | 90.9-95.5  +/-4.0-8.8 | 97.0 +/-6.9 | 93.4 +/-6.9 |
| Arterial SO_2_  post-correction [%] | 94.8 +/-8.7 | 98 +/-10.1 | 97.6 +/-1.67 |  |  |  |  |  |
| Venous SO_2_  pre-correction [%] | 43.7 +/-11.5 | Blue eyes: 74.1 +/-6.6  Green eyes: 65.4 +/-11.1  Brown eyes: 61.7 +/-9.9 |  | 55.6 +/-6.3 | 56.9 +/-6.3 | 53.3-58.6  +/-6-11.1 | 52.8 +/-8.3 | 54.2 +/-6.9 |
| Venous SO_2_  post-correction [%] | 56.3 +/-7.0 | 65 +/-11.7 | 55 +/-3.38 |  |  |  |  |  |

*Supplemental Table 2: SO2 results determined by various publications which have investigated influence of retinal pigmentation on SO2.*
